# Supplementary material for: Comparing spatial regression to random forests for large environmental data sets
Source: PLoS One. 2020 Mar 23;15(3):e0229509. doi: 10.1371/journal.pone.0229509 (PMC7089425; doi:10.1371/journal.pone.0229509)
Supplement: S1 File — This supplement contains: (1) a step-by-step description of the covariate selection procedure; (2) computation details of the random forest regression-kriging computations; and (3) additional tables and figures. (PDF) [file pone.0229509.s001.pdf]

Supplement to "Comparing Spatial Regression to Random Forests for Large Environmental Data Sets"

S1 Covariate Selection Procedure

The procedure we used to select a SLM for MMI with StreamCat covariates:

1. Fit an LM using the full set of covariates.
2. Use the AIC to select an LM with a subset of the covariates using a backwards stepwise algorithm (i.e., the `step()` function from R Core Team (2016)).
3. Fit an SLM with the covariates selected for the LM in the previous step. Use ML estimation with reduced rank method.
4. Remove the covariate in the SLM with the largest absolute t-statistic (for the coefficient) and then re-estimate the SLM using the reduced rank method. Continue to remove covariates from the SLM, one at a time, until the AIC of the SLM increases by a significant margin. Select the most parsimonious SLM with AIC score within 2 points of the minimum. An illustration of this process is provided in Figure S1.
5. Fit an SLM with the variables selected in previous step. Use REML estimation with the full-rank covariance matrix.

In steps 3 and 4 we used ML to estimate the SLM since this allowed use of the AIC; however, REML was used to estimate the final model in step 5. For the reduced rank method we used 300 knots evenly spaced across the CONUS. In preliminary analyses, we also found that approximately 100 knots were necessary for parameter estimates to coverage using `optim()`, and that with 300 knots the cross-validation RMSPE was only sightly less than the full-rank model. Also note that the reduced rank method was only used to speed-up estimation during covariate selection (steps 3 and 4) since the final SLM (step 5) was estimated with the full-rank covariance matrix.

To deal with potential collinearity issues, we used the `findCorrelation()` function from the `caret` package of Kuhn (2016) to reduce the pairwise correlations between covariates below a threshold of 0.75. This function screened out 100 of the 209 StreamCat covariates before application of the stepwise selection procedure described above. Thus, to fit the initial LM in step 1 we used 109 StreamCat covariates as well as the ecoregion dummy variables. Note that for the LASSO model we did not initially screen out correlated covariates, and so all 209 covariates were used when estimating a LASSO model with the `glmnet` package.

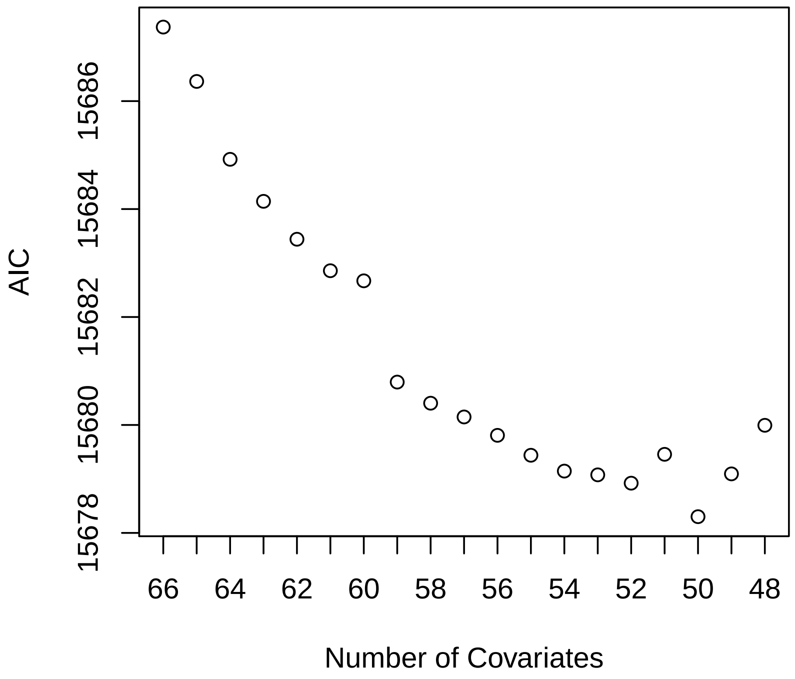

Figure S1: Covariate selection for the SLM with transformations (step 4). The initial SLM was estimated with the 66 covariates that were selected for the LM (step 3). The covariates with the largest absolute t-statistics were then removed one at a time until the AIC increased significantly. The selected SLM contained 48 covariates and had an AIC of 15679.99. Note that the model with 50 covariates attained the minimum AIC value of 15678.3, however models with an AIC difference within 2 points are not significantly different (Burnham and Anderson, 2002); thus, due to the large number of covariates, we selected the more parsimonious model.

S2 Random Forest Regression Kriging Computations

Let $\boldsymbol{Y} - \hat{\boldsymbol{Y}}_{RF} = \boldsymbol{e}' = (e(\boldsymbol{s}_1), \cdots, e(\boldsymbol{s}_n))'$ be a random vector of residuals, where $\hat{\boldsymbol{Y}}_{RF}$ are the RF predictions of $\boldsymbol{Y}$. Assume that $E(e(\boldsymbol{s}_i)) = 0$ and $\text{cov}(\boldsymbol{e}) = \boldsymbol{\Sigma}$; also assume an exponential covariance model such that the (i, j) entry of $\boldsymbol{\Sigma}$ is given by $C(\boldsymbol{s}_i, \boldsymbol{s}_j) = \sigma_z^2 \exp(-\|\boldsymbol{s}_i - \boldsymbol{s}_j\|/\alpha) + I(i = j)\sigma_\epsilon^2$, where $\boldsymbol{\theta} = (\sigma_\epsilon, \sigma_z, \alpha)$ are unknown parameters (nugget, partial sill, and range). Then, for a given realization of the residuals, the negative log-likelihood is given by

$$l(\boldsymbol{\theta}) = 0.5\{n \log(2\pi) + \log(|\boldsymbol{\Sigma}|) + \boldsymbol{e}'\boldsymbol{\Sigma}^{-1}\boldsymbol{e}\}.$$

ML estimates $\hat{\boldsymbol{\theta}}$ are found by minimizing the negative log-likelihood with respect to $\boldsymbol{\theta}$. Note that, in practice, we use the RF out-of-bag predictions from the `randomForest` package (Liaw and Wiener, 2002) to compute the vector of predicted values, $\hat{\boldsymbol{Y}}_{RF}$, at observed locations $\boldsymbol{s}_1, \cdots, \boldsymbol{s}_n$. Also, note that we use the full-rank covariance matrix for ML estimation.

Once ML estimates for the covariance parameters are obtained, spatial predictions for the residuals can be computed using simple kriging (Cressie, 1993, p. 110; Cressie and Wikle, 2011, pp. 136–139). Under the zero-mean assumption, the simple-kriging predictor of the residual at a new location $\boldsymbol{s}_0$ is given by $\hat{e}(\boldsymbol{s}_0) = \boldsymbol{c}'\boldsymbol{\Sigma}^{-1}\boldsymbol{e}$, where $\boldsymbol{c}' = (C(\boldsymbol{s}_0, \boldsymbol{s}_1), \cdots, C(\boldsymbol{s}_0, \boldsymbol{s}_n))$. The simple-kriging variance (minimized mean-square-prediction error) is also given by $\text{var}(\hat{e}(\boldsymbol{s}_0)) = C(\boldsymbol{s}_0, \boldsymbol{s}_0) - \boldsymbol{c}'\boldsymbol{\Sigma}^{-1}\boldsymbol{c}$; note that $C(\boldsymbol{s}_0, \boldsymbol{s}_0) = \sigma_z^2 + \sigma_\epsilon^2$ is commonly referred to as the sill. Then the RFRK prediction is $\hat{Y}(\boldsymbol{s}_0) = \hat{Y}_{RF}(\boldsymbol{s}_0) + \hat{e}(\boldsymbol{s}_0)$ and 90% prediction interval is $\hat{Y}(\boldsymbol{s}_0) \pm 1.645\sqrt{\text{var}(\hat{e}(\boldsymbol{s}_0))}$, where $\hat{Y}_{RF}(\boldsymbol{s}_0)$ is the RF prediction at s_0.

S3 Additional Figures and Tables

Table S1: Regression coefficient summary for the SLM with transformations. Estimated Box-Cox transformations parameters λ_1 (exponent) and λ_2 (shifting) are also shown. Note that transformed covariates were standardized before fitting the model (subtracted mean and divided by standard deviation). Top 5 covariates, ranked in terms of absolute t-statistics, are in bold face.

	λ_1	λ_2	Est.	SE	t	p-val.
Intercept			55.79	3.06	18.24	2.42e-68
NAP			-6.08	3.28	-1.85	6.43e-02
NPL			10.31	3.15	3.28	1.07e-03
**SAP**			-12.56	2.18	-5.77	9.34e-09
TPL			5.61	2.28	2.46	1.38e-02
**WMT**			-19.77	2.92	-6.78	1.65e-11
XER			-5.34	2.91	-1.83	6.68e-02
AvgTmaxCat_BC	0.1	0	-2.07	0.94	-2.20	2.77e-02
**AvgWetIndxCat_BC**	0.0	0	-3.82	0.63	-6.04	1.89e-09
AvgWetIndxWs_BC	0.0	0	-3.70	0.80	-4.65	3.51e-06
CanalDensCat_Bin			3.57	1.73	2.06	3.95e-02
CBNFWs_BC01	1.3	0	0.78	0.45	1.73	8.36e-02
ClayCat_BC2	0.0	1	-1.34	0.72	-1.85	6.40e-02

FertCat_BC01	0.0	1e-10	1.01	0.59	1.72	8.60e-02
FertWs_Bin			-3.95	1.92	-2.06	3.95e-02
MineDensWsRp100_BC01	1.1	0	3.25	0.88	3.71	2.13e-04
NABD_NrmStorWs_Bin			-3.89	1.35	-2.89	3.94e-03
NABD_NrmStorWs_BC01	0.3	0	-1.54	0.60	-2.55	1.09e-02
NH4Cat_BC	1.3	0	-1.46	0.99	-1.48	1.40e-01
NPDESDensWs_BC01	0.1	0	-2.01	0.83	-2.43	1.53e-02
OmCat_BC	0.3	0	-1.58	0.63	-2.50	1.25e-02
OmWs_BC	0.0	0	2.18	0.75	2.90	3.81e-03
PctAg2006Slp10Cat_BC01	0.2	0	-1.17	0.68	-1.72	8.65e-02
PctAg2006Slp20Ws_BC01	0.0	0.01	-1.50	0.75	-2.01	4.50e-02
PctCrop2006CatRp100_Bin			-2.15	1.01	-2.14	3.27e-02
PctCrop2006CatRp100_BC01	2.0	0	-1.87	0.71	-2.62	8.90e-03
PctFrstLoss06_09Cat_BC01	0.0	1e-10	-1.60	0.67	-2.38	1.73e-02
PctFrstLossWsRp100_Bin			5.45	1.46	3.73	2.00e-04
PctGlacLakeFineWs_BC01	1.8	0	-2.30	0.98	-2.35	1.89e-02
PctGlacTilCrsWs_BC01	0.0	0.1	-5.57	1.46	-3.82	1.36e-04
PctHbWet2006Cat_BC01	3.0	0	-1.59	0.66	-2.39	1.71e-02
PctImp2006CatSlp10_BC01	0.4	0	-2.01	0.68	-2.96	3.09e-03
PctNonCarbResidCat_Bin			-2.46	1.08	-2.29	2.21e-02
PctUrbHi2006Cat_Bin			-2.43	1.49	-1.63	1.03e-01
PctUrbLo2006WsRp100_Bin			-4.45	1.38	-3.24	1.23e-03
PctUrbMd2006WsRp100_Bin			4.11	1.35	3.06	2.28e-03
PctUrbMd2006WsRp100_BC01	0.1	0	-1.88	0.66	-2.83	4.69e-03
PctWdWet2006CatRp100_BC01	0.0	1e-10	1.62	0.54	3.00	2.73e-03
PermCat_BC	0.0	1	6.95	2.00	3.47	5.36e-04
PermCat_BC2	0.0	1	-7.02	2.08	-3.38	7.41e-04
Pestic97Ws_Bin			-9.07	2.48	-3.66	2.61e-04
Pestic97Ws_BC01	0.0	0.6	-2.05	0.72	-2.85	4.47e-03
RdCrsSlpWtdCat_BC01	0.0	1e-10	2.46	0.70	3.51	4.63e-04
RdDensCatRpBf100_Bin			-4.13	1.31	-3.15	1.68e-03
RdDensCatRpBf100_BC01	1.8	0	-0.98	0.52	-1.88	5.97e-02
RunoffCat_BC	0.0	0.2	4.35	0.77	5.64	1.97e-08
**WsAreaSqKm_BC**	0.0	0	22.12	2.02	10.97	3.82e-27
**WsAreaSqKm_BC2**	0.0	0	-20.27	1.89	-10.71	5.17e-26

NOTE: The tags at the end of the covariates names indicate the type of transformation: 'BC' indicates Box-Cox transformation $g(x, \lambda_1, \lambda_2)$, 'BC2' indicates a quadratic transformation $(g(x, \lambda_1, \lambda_2))^2$, 'Bin' indicates a zero/nonzero dummy variable $I(x \neq 0)$, and 'BC01' indicates the interaction $g(x, \lambda_1, \lambda_2)I(x \neq 0)$. The types of transformations are described in detail in Section 2.2 of the paper. The spatial regression model also includes dummy variables for the following ecoregions: Northern Appalachians (NAP), Northern Plains (NPL), Southern Appalachians (SAP), Temperate Plains (TPL), Westerm Mountain (WMT), and Xeric (XER). StreamCat covariate descriptions are provided in Table S2.

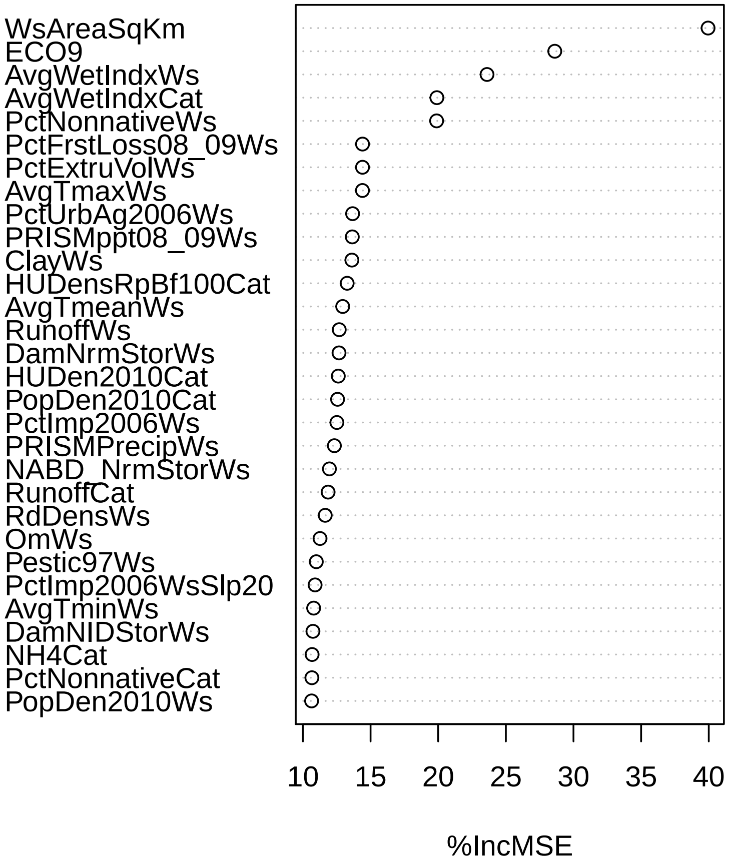

Figure S2: Variable importance plot for random forest model with top 30 predictor variables. Variable importances were computed using the `importance()` function from the `randomForest` package and setting the argument `type=1`. This gives the permutation-based measure (increase in MSE when each variable is permuted in the out-of-bag data). StreamCat covariate descriptions are provided in Table S2.

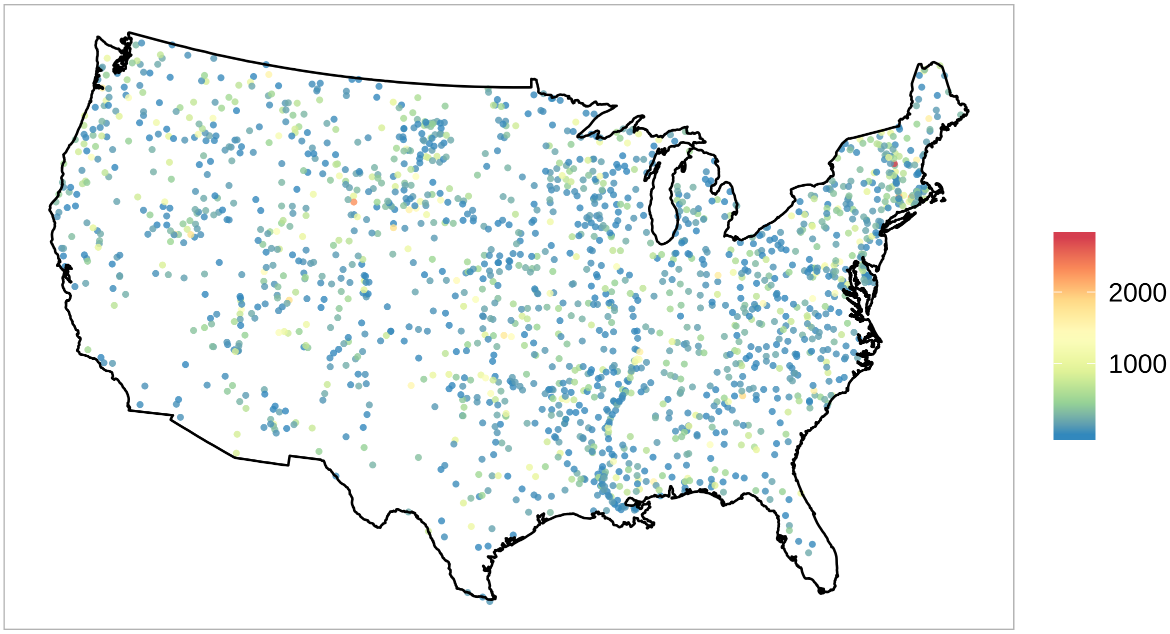

Figure S3: Map of the squared residuals from the SLM with covariate transformations. Residuals were computed as $\hat{\boldsymbol{e}} = \boldsymbol{Y} - \boldsymbol{X}\hat{\boldsymbol{\beta}}$, where $\hat{\boldsymbol{\beta}} = (\boldsymbol{X}'\boldsymbol{\Sigma}^{-1}\boldsymbol{X})^{-1}\boldsymbol{X}'\boldsymbol{\Sigma}^{-1}\boldsymbol{Y}$ are the generalized least squares estimates.

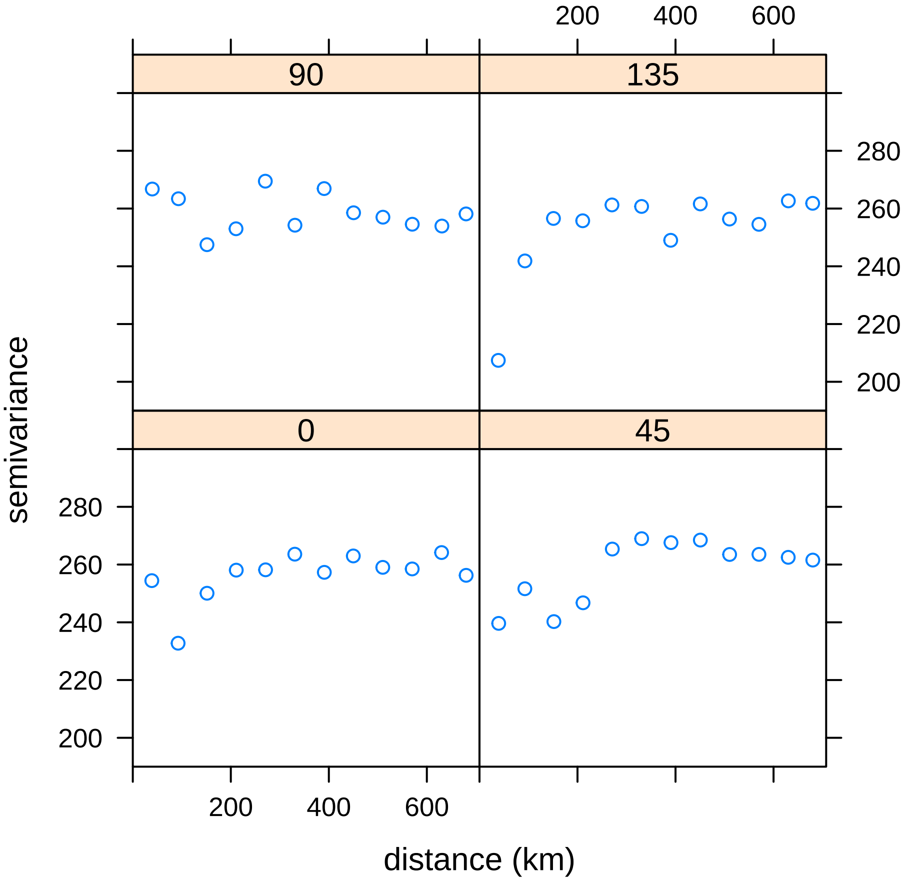

Figure S4: Directional semivariograms of the residuals from the SLM with covariate transformations. The plot was created using the R package `gstat` (Gräler et al., 2016).

Table S2: Descriptions of StreamCat covariates shown in the spatial regression summary (Table S1) and RF variable importance plot (Figure S2). Further details about the StreamCat data set can be found at `ftp://newftp.epa.gov/EPADataCommons/ORD/NHDPlusLandscapeAttributes/StreamCat/WelcomePage.html`.

Covariate Name	Description
AvgTmaxCat	PRISM climate data - 30-year normal maximum temperature (C): Annual period: 1981-2010 within the catchment
AvgTmaxWs	PRISM climate data - 30-year normal maximum temperature (C): Annual period: 1981-2010 within the watershed
AvgTmeanWs	PRISM climate data - 30-year normal mean temperature (C): Annual period: 1981-2010 within the watershed
AvgTminWs	PRISM climate data - 30-year normal minimum temperature (C): Annual period: 1981-2010 within the watershed
AvgWetIndxCat	Mean topographic (30m DEMs) wetness index (https://en.wikipedia.org/wiki/Topographic_Wetness_Index) within the catchment
AvgWetIndxWs	Mean topographic (30m DEMs) wetness index (https://en.wikipedia.org/wiki/Topographic_Wetness_Index) within the watershed
CanalDensCat	Density of NHDPlus line features classified as canal, ditch, or pipeline within the catchment (km/ square km)
CBNFWs	Mean crop biological nitrogen fixation within the upstream watershed
ClayCat	Mean % clay content of soils (STATSGO) within catchment
ClayWs	Mean % clay content of soils (STATSGO) within watershed
DamNIDStorWs	Volume all reservoirs (NID_STORA in NID) per unit area of watershed (cubic meters/square km)
DamNrmStorWs	Volume all reservoirs (NORM_STORA in NID) per unit area of watershed (cubic meters/square km)
FertCat	Mean rate of synthetic nitrogen fertilizer application to agricultural land in kg N/ha/yr, within the catchment
FertWs	Mean rate of synthetic nitrogen fertilizer application to agricultural land in kg N/ha/yr, within watershed
HUDen2010Cat	Mean housing unit density (housing units/square km) within catchment
HUDensRpBf100Cat	Mean housing unit density (housing units/square km) within catchment and within 100-m buffer of NHD stream lines
MineDensWsRp100	Density of mines sites within watershed and within 100-m buffer of NHD stream lines (mines/square km)
NABD_NrmStorWs	Volume all reservoirs (NORM_STORA in NID) per unit area of watershed (cubic meters/square km)
NH4Cat	Annual gradient map of precipitation-weighted mean deposition for ammonium ion concentration wet deposition in kg of NH4/ha/yr, within catchment
NPDESDensWs	Density of permitted NPDES (National Pollutant Discharge Elimination System) sites within watershed (sites/square km)

OmCat	Mean organic matter content (% by weight) of soils (STATSGO) within catchment
OmWs	Mean organic matter content (% by weight) of soils (STATSGO) within watershed
PctAg2006Slp10Cat	% of catchment area classified as ag land cover (NLCD 2006 classes 81-82) occurring on slopes > 10%
PctAg2006Slp20Ws	% of catchment area classified as ag land cover (NLCD 2006 classes 81-82) occurring on slopes > 20%
PctCrop2006CatRp100	% of catchment area classified as crop land use (NLCD 2006 class 82) within a 100-m buffer of NHD streams
PctExtruVolWs	% of watershed area classified as as lithology type: extrusive volcanic rock
PctFrstLoss06_09Cat	% of catchment area that experienced forest loss (yrs. 2006-2009)
PctFrstLoss08_09Ws	% of watershed area that experienced forest loss (yrs. 2008-2009)
PctFrstLossWsRp100	% of watershed area that experienced forest loss (all years) within 100-m buffer of NHD stream lines
PctGlacLakeFineWs	% of watershed area classified as as lithology type: glacial lake sediment, fine-textured
PctGlacTilCrsWs	% of watershed area classified as as lithology type: glacial till, coarse-textured
PctHbWet2006Cat	% of catchment area classified as herbaceous wetland land cover (NLCD 2006 class 95)
PctImp2006CatSlp10	Mean imperviousness of anthropogenic surfaces (NLCD 2006) within catchment occuring on slopes > 10%
PctImp2006Ws	Mean imperviousness of anthropogenic surfaces (NLCD 2006) within watershed
PctImp2006WsSlp20	Mean imperviousness of anthropogenic surfaces (NLCD 2006) within catchment occuring on slopes > 20%
PctNonCarbResidCat	% of catchment area classified as lithology type: non-carbonate residual material
PctNonnativeCat	% of catchment area classified as non-native vegetation based on LandFire classes (http://www.landfire.gov/)
PctNonnativeWs	% of watershed area classified as non-native vegetation based on LandFire classes (http://www.landfire.gov/)
PctUrbAg2006Ws	% of watershed area classified as urban and agricultural land uses (NLCD 2006 classes 21-24, 81-82) NHD stream lines
PctUrbHi2006Cat	% of catchment area classified as developed, high-intensity land use (NLCD 2006 class 24)
PctUrbLo2006WsRp100	% of watershed area classified as developed, low-intensity land use (NLCD 2006 class 22) within a 100-m buffer of NHD streams
PctUrbMd2006WsRp100	% of watershed area classified as developed, medium-intensity land use (NLCD 2006 class 23) within a 100-m buffer of NHD streams
PctWdWet2006CatRp100	% of catchment area classified as woody wetland land cover (NLCD 2006 class 90) within a 100-m buffer of NHD streams
PermCat	Mean permeability (cm/hour) of soils (STATSGO) within catchment
Pestic97Ws	Mean pesticide use (kg/km2) in yr. 1997 within watershed

PopDen2010Cat	Mean populating density (people/square km) within catchment
PopDen2010Ws	Mean populating density (people/square km) within watershed
PRISMppt08_09Ws	PRISM climate data - mean precipitation (mm): Annual period: 2008-2009 within the watershed
PRISMPrecipWs	PRISM climate data - 30-year normal mean precipitation (mm): Annual period: 1981-2010 within the watershed
RdCrsSlpWtdCat	Density of roads-stream intersections (2010 Census Tiger Lines-NHD stream lines) multiplied by NHDPlusV21 slope within catchment (crossings*slope/square km)
RdDensCatRpBf100	Density of roads (2010 Census Tiger Lines) within catchment and within a 100-m buffer of NHD stream lines (km/square km)
RdDensWs	Density of roads (2010 Census Tiger Lines) within watershed (km/square km)
RunoffCat	Mean runoff (mm) within catchment
RunoffWs	Mean runoff (mm) within watershed
WsAreaSqKm	Watershed area (square km) at NHDPlus stream segment outlet, i.e., at the most downstream location of the vector line segment

References

Burnham, K. P. and Anderson, D. R. (2002). *Model selection and multimodel inference: a practical information-theoretic approach.* Springer-Verlag New York, second edition.

Cressie, N. (1993). *Statistics for spatial data.* John Wiley & Sons.

Cressie, N. and Wikle, C. K. (2011). *Statistics for spatio-temporal data.* John Wiley & Sons.

Gräler, B., Pebesma, E., and Heuvelink, G. (2016). Spatio-temporal interpolation using gstat. *The R Journal*, 8:204–218.

Kuhn, M. (2016). *caret: Classification and Regression Training.* Contributions from Jed Wing, Steve Weston, Andre Williams, Chris Keefer, Allan Engelhardt, Tony Cooper, Zachary Mayer, Brenton Kenkel, the R Core Team, Michael Benesty, Reynald Lescarbeau, Andrew Ziem, Luca Scrucca, Yuan Tang and Can Candan. R package version 6.0-71.

Liaw, A. and Wiener, M. (2002). Classification and regression by randomForest. *R news*, 2(3):18–22.

R Core Team (2016). *R: A Language and Environment for Statistical Computing.* R Foundation for Statistical Computing, Vienna, Austria.
