# Supplementary material for: Comparing spatial regression to random forests for large environmental data sets
Source: PLoS One. 2020 Mar 23;15(3):e0229509. doi: 10.1371/journal.pone.0229509 (PMC7089425; doi:10.1371/journal.pone.0229509)
Supplement: S1 Data and Code — Link to GitHub repository: https://github.com/ericwfox/slmrf. (DOCX) [file pone.0229509.s002.docx]

**S1 Data and Code. R package with data sets and simulation code.** Link to GitHub repository:

https://github.com/ericwfox/slmrf.
